# Silent Damage by Micro-aspirations: Untangling the Connection of Gastroesophageal Reflux Disease (GERD) and Achalasia With Interstitial Lung Disease

**DOI:** 10.7759/cureus.70113

**Published:** 2024-09-24

**Authors:** Mahrukh A Khan, Muhammad Saad Anwar, Alina Nayyar, Fatima Kausar Nawaz, Doantrang Du

**Affiliations:** 1 Pulmonary and Critical Care Medicine, SUNY Upstate Medical University, Syracuse, USA; 2 Internal Medicine, Monmouth Medical Center, Long Branch, USA; 3 Internal Medicine, King Edward Medical University, Lahore, PAK; 4 Medicine, Wright Center for GME, Scranton, USA; 5 Internal Medicine, RWJBarnabas Health, Long Branch, USA

**Keywords:** achalasia, acid reflux, gerd, ild interstitial lung disease, microaspiration

## Abstract

Gastroesophageal reflux disease (GERD) frequently triggers respiratory conditions such as asthma and pneumonia. Inflammation occurs as a result of aspirated material, leading to symptoms such as cough, sputum production, chest discomfort from the involvement of the lower respiratory tract, and voice hoarseness owing to the involvement of the larynx. Repeated exposure to irritants can lead to fibrosis in the lungs. However, little is known about the association of achalasia with interstitial lung disease (ILD). We present a case of a patient with GERD who presented with cough and reflux for three months. Extensive testing confirmed the diagnosis of achalasia, and pneumatic dilation provided relief. The patient returned after two years with additional symptoms of shortness of breath. A CT scan of the chest showed worsening reticular changes and ground-glass opacity, indicative of ILD. An unremarkable toxin exposure history and a negative autoimmune panel led clinicians to explore the possible relationship between achalasia and ILD, highlighting the need for further exploration and research in this area.

## Introduction

Aspiration is the inhalation of the oropharyngeal or gastric content into the upper and lower respiratory tract. Nearly 50% of healthy individuals may aspirate small amounts of oropharyngeal content during sleep, as the gastrointestinal and respiratory tracts have a common origin, and there is a complex interplay of breathing and swallowing. However, healthy individuals have mechanisms to prevent aspiration, such as the cough reflex, glottis closure, and functional esophageal sphincter [1,2]. Studies have shown that disorders of the oropharyngeal, esophageal, gastric region, and even neurodegenerative disorders can contribute to the development of laryngotracheal and pulmonary disorders due to aspiration, including vocal cord dysfunction, asthma, aspiration pneumonitis or pneumonia, interstitial lung disease (ILD), chronic bronchiolitis obliterans, and idiopathic pulmonary fibrosis [3]. While gastroesophageal reflux disease (GERD) is known to have a higher prevalence of chronic respiratory disease, little is understood about the role of achalasia, a neurodegenerative motility disorder of the esophagus that leads to nonrelaxation of the lower esophageal sphincter (LES). Despite being on the opposite spectrum of LES dysfunction, both GERD and achalasia can coexist and theoretically contribute to the development of chronic respiratory diseases [4]. Herein, we present a case of a 65-year-old female with a chronic history of GERD who visited the clinic with complaints of dry cough and severe reflux. Further testing led to a diagnosis of ILD due to underlying achalasia in the background of GERD.

## Case presentation

A 65-year-old woman with a history of GERD was evaluated in the pulmonology clinic around three years ago due to persistent dry cough, occasional hoarseness, and worsening reflux for the past three months. She had been non-compliant with follow-up appointments. The patient had no history of allergies, asthma, pet sensitivity, extensive travel, smoking, vaping, recreational drug use, exposure to tuberculosis, or toxic chemicals. She had a low appetite and unintentional weight loss and denied any episodes of hemoptysis, pneumonia, or bronchitis. She occasionally used antacids and did not have any family history of malignancy. Her BMI was 24; upon evaluation, she was stable and saturating 93% on room air. A pulmonary exam revealed mild, fine crackles on lung bases.

**Figure 1:**
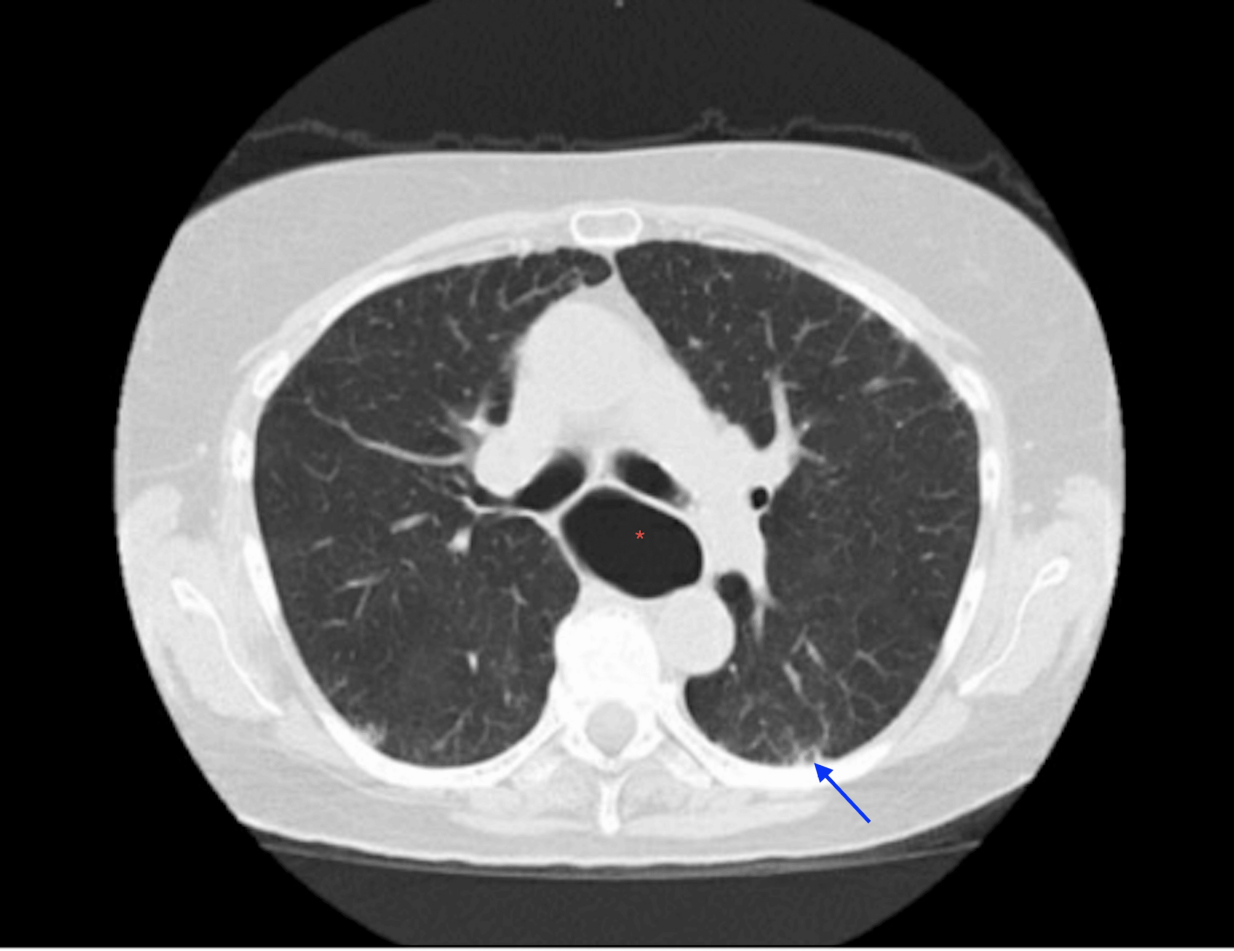
Mild bibasilar reticular changes (blue arrow) with esophageal dilatation (red asterisk)

After extensive counseling, the patient was agreeable to further investigation to rule out the causes of cough and hoarseness. The complete blood count was within normal limits, including eosinophil counts. Autoimmune workup was ordered, including ANA, anti-dsDNA, anti-smith, rheumatoid factor, anti-CCP, anti-Ro (SS-A), anti-La (SS-B), anti-Scl-70, anti-topoisomerase, anti-RNA polymerase, and anti-U1-RNP, which turned out to be negative. Sputum cultures and respiratory viral panels remained negative. A chest computed tomography (CT scan) was obtained, demonstrating mild reticular changes in the basilar region. A dilated esophagus was also noted (Figure 1). IgM and IgG antibodies of *Trypanosoma cruzi *were also undetectable.

**Figure 2:**
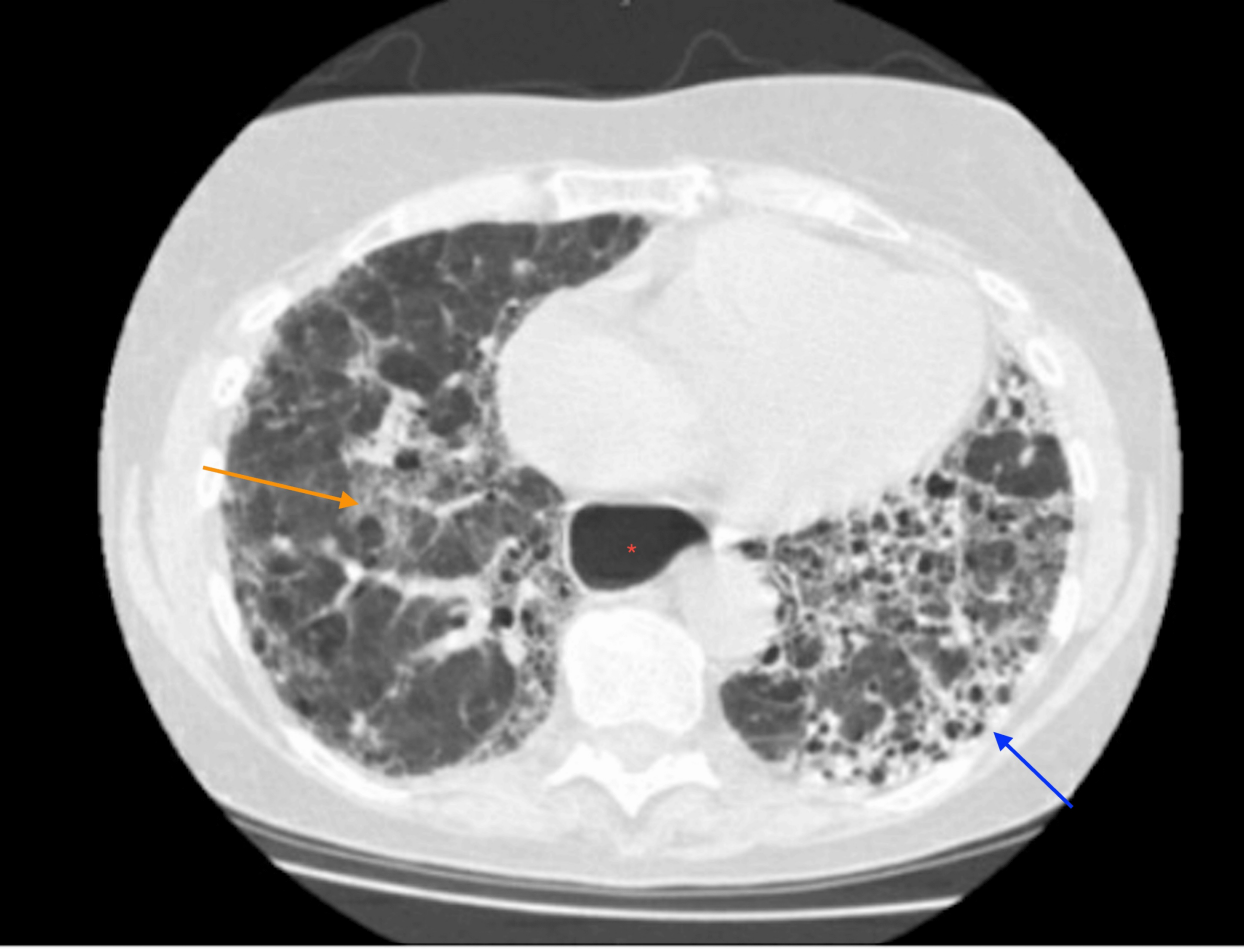
Extensive reticular, fibrotic changes (blue arrow), and ground glass opacity (orange arrow) consistent with findings of interstitial lung disease. Note persistent dilated esophagus (red asterisk)

She was referred to the gastroenterologist for further investigation. The patient did not agree to undergo a barium swallow study as she did not want to drink barium contrast. Hence, esophageal manometry was performed, which demonstrated LES's integrated relaxation pressure (IRP) of 17 mmHg with premature contractions for 25% of the swallows without normal peristalsis, confirming the diagnosis of achalasia. Pneumatic dilatation was carried out, resulting in improved dysphagia and chronic cough. The patient was advised to follow strict aspiration precautions and prescribed anti-reflux medications. However, the patient was lost to follow-up and returned after two years with worsening dry cough, shortness of breath, and worsening reflux. A CT scan of the chest revealed diffuse reticular changes and bilateral ground glass opacities (GGOs), consistent with findings of ILD alongside the persistence of esophageal dilation (Figure 2).

The patient denied any irritant exposure. Sputum culture, respiratory viral panel, and autoimmune profile remain unremarkable like before. Although a heller myotomy was discussed, the patient continued to refuse surgical intervention. Pneumatic dilation was repeated for symptomatic relief. Nintedanib 150 mg twice daily was initiated, along with a referral to a pulmonary rehabilitation center to improve the patient's quality of life (QoL) and slow the progression of fibrotic changes. The patient experienced several hospitalizations due to respiratory infections in the past year. After six months of follow-up, the patient reported minimal improvement in quality of life.

## Discussion

Achalasia is a primary neurodegenerative esophageal disorder characterized by the absence of esophageal peristalsis and LES relaxation. It is caused by damage to the myenteric plexus, hindering inhibitory neurons from coordinating peristalsis. It affects both sexes and all ages, with a prevalence rate of around 27.1 per 100,000 people. Primary achalasia is mostly idiopathic and prevalent everywhere except South America, where secondary achalasia associated with Chagas disease caused by *T. cruzi* is endemic [5,6]. However, the antibodies of *T. cruzi* were negative in our patient. Achalasia presents as dysphagia of both liquid and solid food content, along with early symptoms such as chest pain, early satiety, heartburn, and regurgitation. Esophageal dilatation proximal to a tight LES leads to the stasis of ingested content, making regurgitation a frequently presented symptom. One study reported that regurgitation was present in 73.3% of achalasia cases, predisposing patients to recurrent micro-aspiration [7]. Heartburn is also noted in 13-68% of patients with achalasia. GERD commonly presents as heartburn and regurgitation due to gastric acid reflux, which often delays the diagnosis of achalasia by 2-7 years [4,6].

Although achalasia and GERD are on opposite ends of the range regarding LES laxity, the causes of regurgitation and heartburn differ. In GERD, acid reflux is the most common causal factor. However, in achalasia, mechanisms of heartburn and regurgitation include esophageal spasms and ingested static irritants in the aperistaltic esophageal region, which can also ferment into lactic acid [4]. However, in some cases, GERD can progress to diffuse esophageal spasm and then transform into achalasia, which can theoretically explain the period of acid reflux and misdiagnosis of achalasia as GERD in the early stages [4,6].

Both GERD and achalasia are noted to be related to the development of chronic respiratory disorders. Gastric fluid aspiration in GERD exposes the airway to acidic pH and pepsin, which exert their cytotoxic effect by activating an acute cytokine cascade, causing alveolitis. In chronic cases of micro-aspiration, fibrotic changes develop in pulmonary alveoli, interstitium, and extracellular matrix by continued activation of transforming growth factor β (TGF-β) [8]. A similar pathogenesis is also hypothesized in achalasia by reflux of food particles and possibly fermented lactic acid. According to research by Makharia et al., 16.6% of patients with achalasia had restrictive pulmonary function test patterns, and the high-resolution CT showed GGOs, fibrosis, and bronchiectasis in roughly 30% of the patients [7]. Parshad et al. reported that 43% of the achalasia patients had parenchymal changes on HRCT. Among these patients, 46.1% of them had acute lung abnormalities like consolidation, GGO, and nodular opacities, while 53.8% had chronic changes like fibrosis, calcified nodules, and air trapping. All these patients underwent Heller's cardiomyotomy. Repeat HRCT at a mean follow-up of one year showed resolution of acute changes, while chronic changes remained unchanged in retested patients [9].

There are several approaches for diagnosing microaspiration. Initially, a detailed history of symptoms is crucial for determining further testing. In our case, given the prominent history of acid reflux and evidence of esophageal dilation on imaging, a barium swallow study was planned, which was refused by our patient. Classically, a diagnosis of achalasia is confirmed with the typical "bird beak" appearance on a barium swallow. Examining the pattern of peristalsis is also essential for identifying dysmotility and spasms, which contribute to reflux. The gold standard of diagnosis of achalasia is esophageal manometry, which classifies achalasia in manometric types based on contractility patterns in the esophageal body. Type I (classic achalasia) is characterized by 100% failed peristalsis. Type II, the most common type, is diagnosed by at least 20% panesophageal pressurization without normal peristalsis. Type III is defined as the presence of premature contraction in at least 20% of the swallows without normal peristalsis. Contractions are considered premature if propagated in less than 4.5 seconds [10]. Our patient had type III achalasia.

Due to the patient's established history of GERD, additional tests such as 24-hour pH monitoring with manometry, pepsin, and bile salt level in bronchoalveolar lavage (BAL) and exhaled breath condensate (EBC) were not conducted [1]. In this case, the absence of autoimmune disorder, connective tissue disorder, smoking history, occupational hazards exposure, prolonged exposure to avian species, exposure to pneumotoxic medications, positive bacterial and fungal culture, allergies, pertinent family history, obesity, and sleep disorder led us to determine chronic microaspiration as the most likely inducing factor for ILD.

Management of achalasia can be approached in various ways, and it's important to consider the patient's preferences and needs. Oral adjuncts like calcium channel blockers are also provided to relax the LES. Botulinum toxin injections can also have variable responses in terms of efficacy and lasting. In our case, the patient chose pneumatic esophageal dilatation over surgical intervention. Surgical interventions such as peroral endoscopic myotomy (POEM) and Heller myotomy lead to symptom improvement in 83% to 100% of cases, with a remission rate of 67%-85%. However, even with anti-reflux procedures, 10-30% of patients will require anti-reflux therapy for increased GERD [6,10].

Managing achalasia is challenging, and it often requires long-term multidisciplinary care. In our case, the evidence of fibrotic changes in our patient led us to initiate the antifibrotic agent nintedanib, a tyrosine kinase inhibitor. Studies have shown that it slows the rate of decline in forced vital capacity (FVC) by 50% over a year, protects against acute exacerbation, and provides survival benefits. Immunomodulators were avoided in our case as autoimmune disorders were ruled out. A lung transplant, often considered a last resort, was not deemed suitable for our patient due to a relapse of achalasia requiring repeated dilation and recent episodes of respiratory infection. The patient was referred to pulmonary rehabilitation; however, due to the progressive nature of the disease, significant improvement in quality of life was not achieved [11].

## Conclusions

Restrictive lung disease presents as a challenging and debilitating condition that necessitates a thorough evaluation to uncover any underlying secondary causes. Although uncommon, gastrointestinal issues like GERD and achalasia can contribute to the development of chronic lung pathologies. The microaspirations resulting from these conditions can trigger lung inflammation, which may progress to lung fibrosis. Therefore, prompt identification through CT scans and timely interventions are crucial for symptom management. Addressing the root causes can significantly slow the progression of disease.
